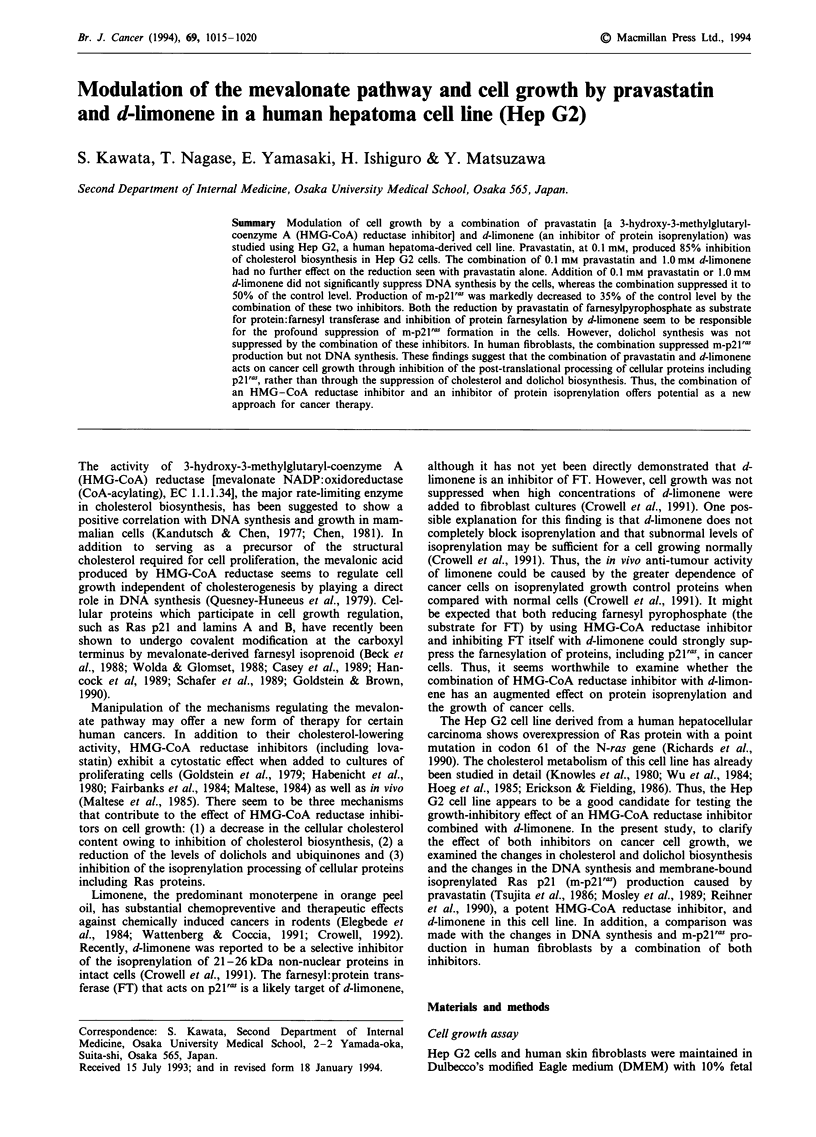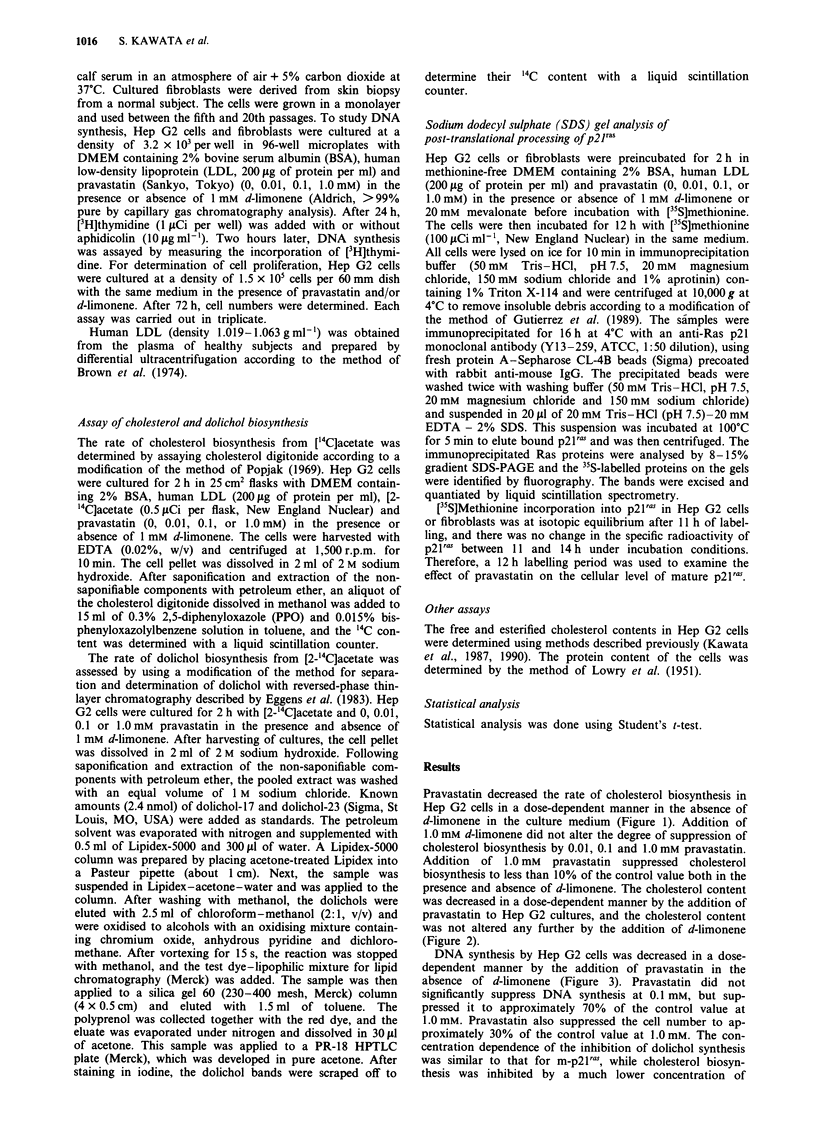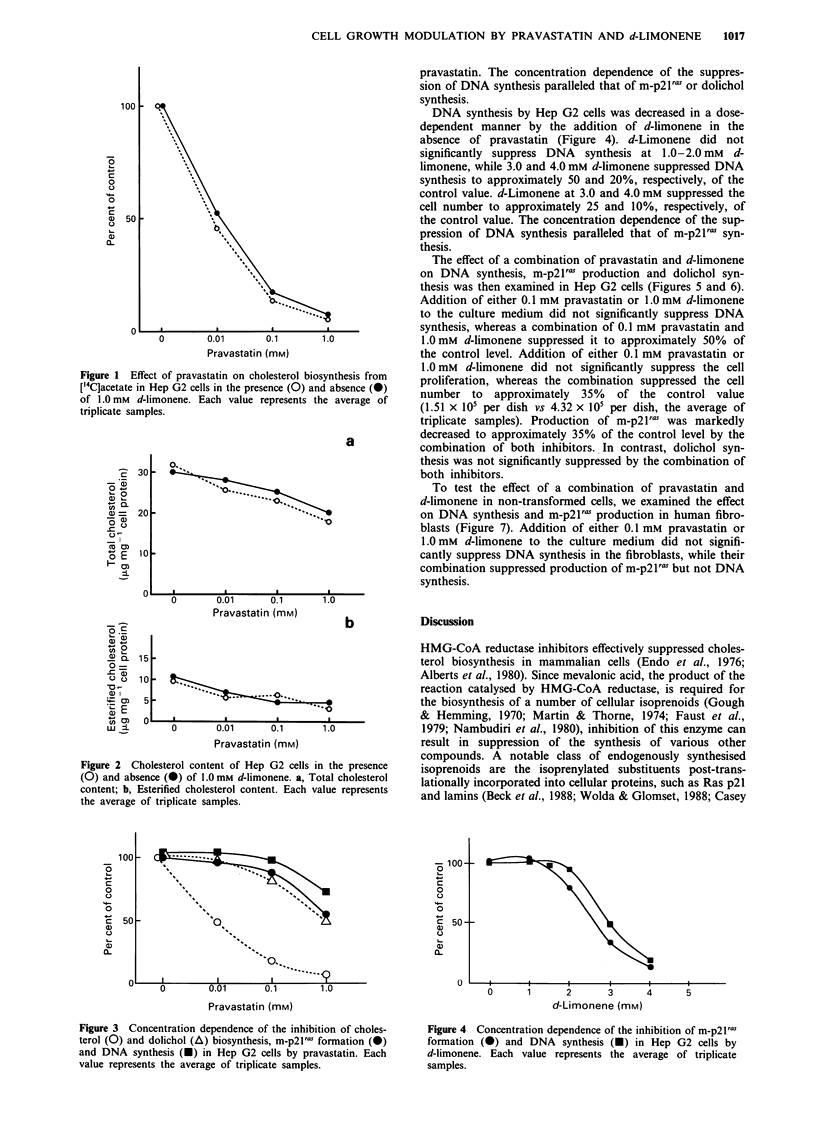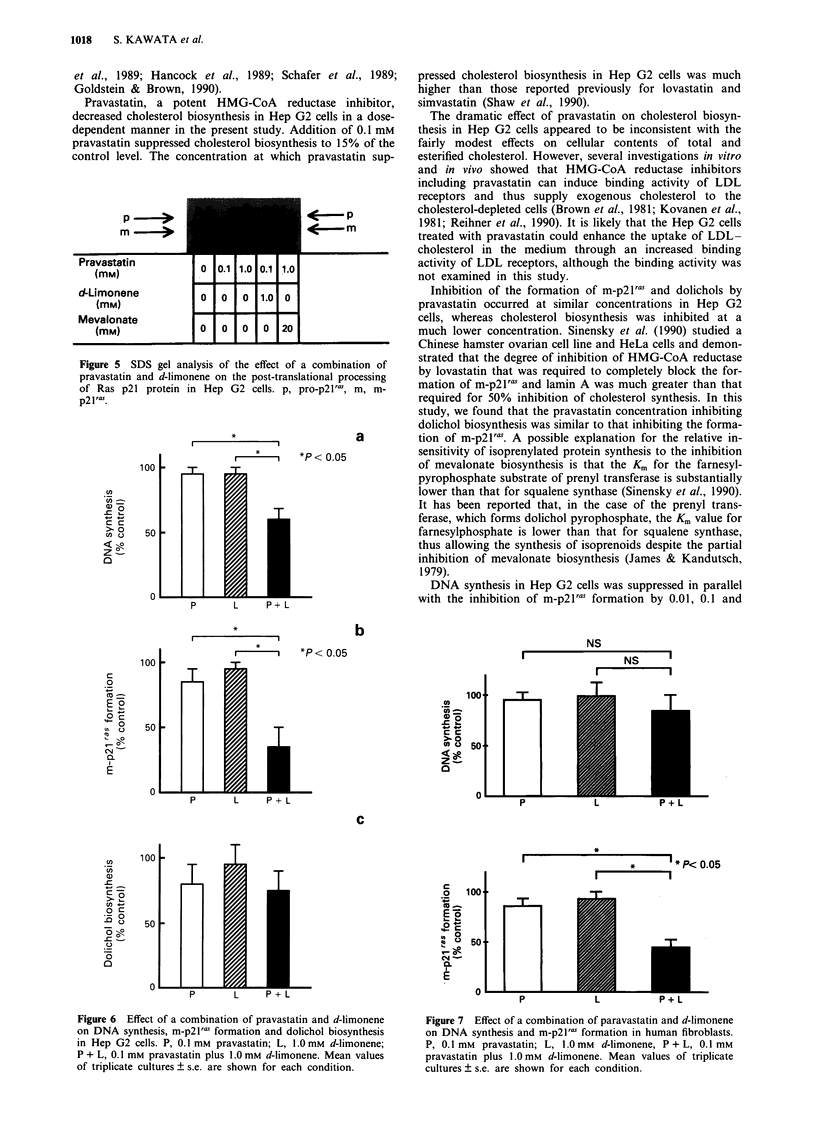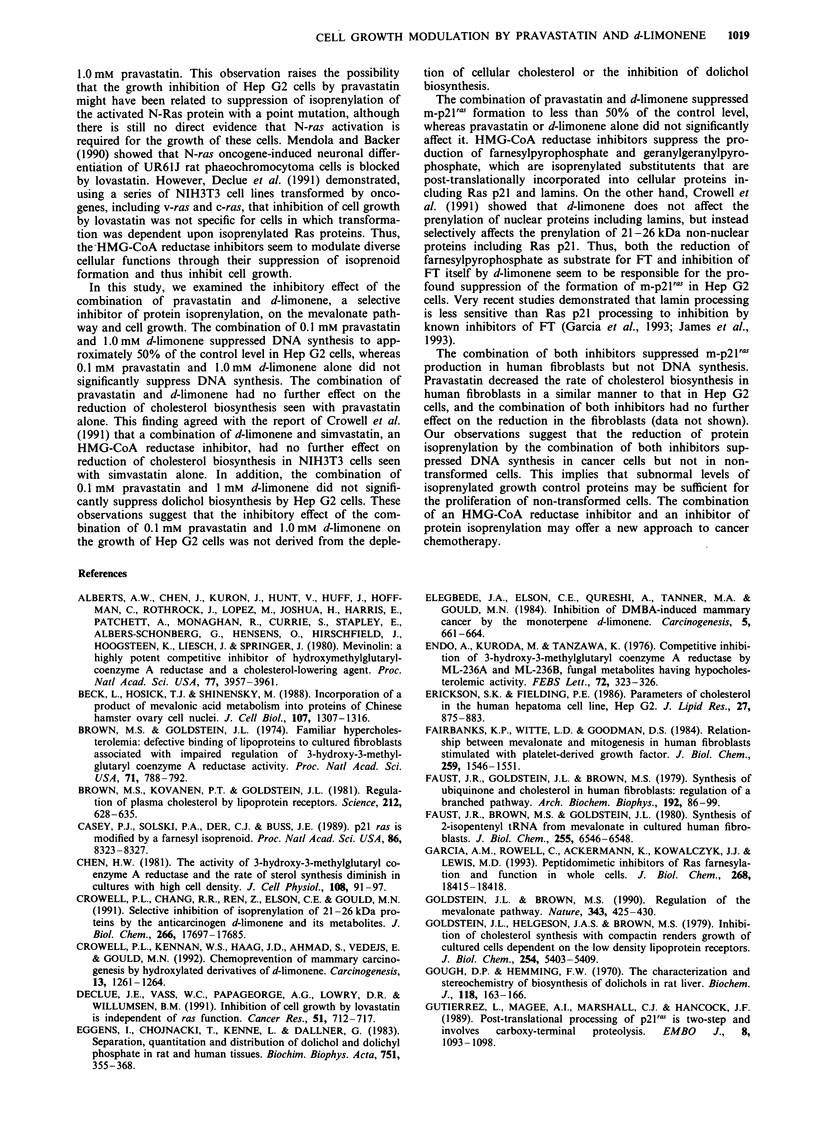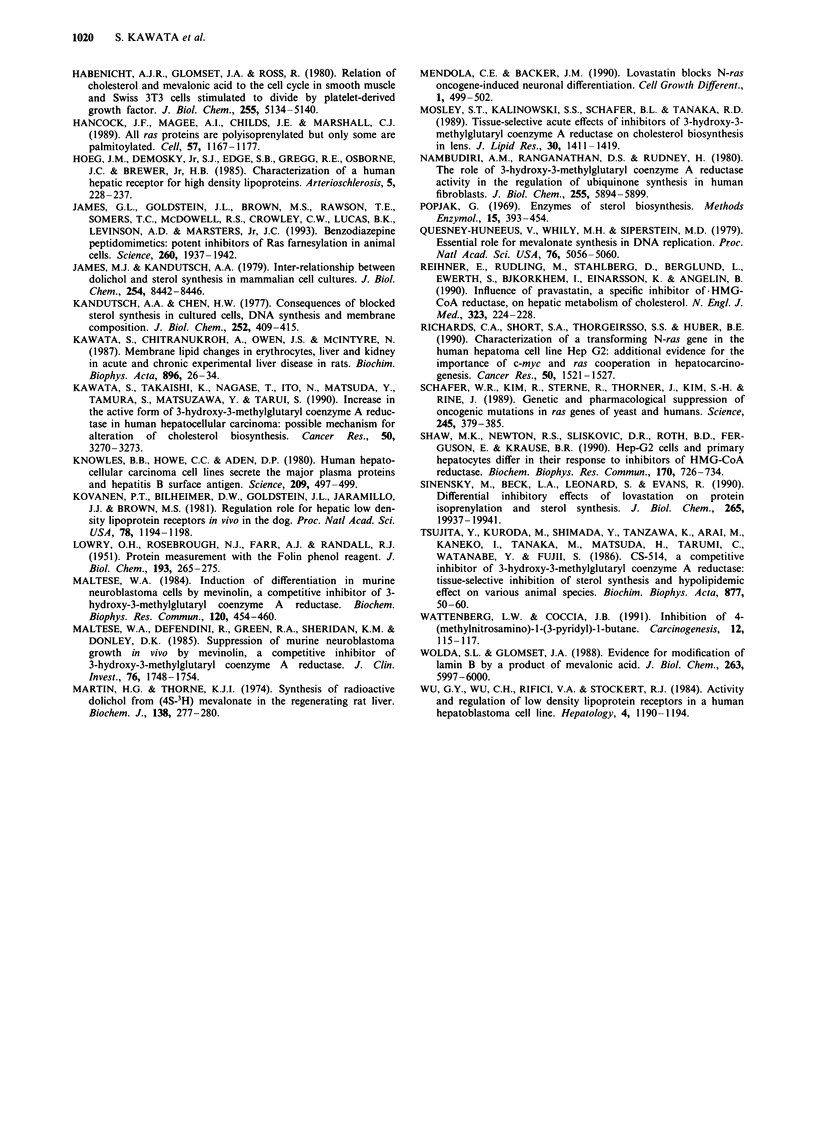# Modulation of the mevalonate pathway and cell growth by pravastatin and d-limonene in a human hepatoma cell line (Hep G2).

**DOI:** 10.1038/bjc.1994.199

**Published:** 1994-06

**Authors:** S. Kawata, T. Nagase, E. Yamasaki, H. Ishiguro, Y. Matsuzawa

**Affiliations:** Second Department of Internal Medicine, Osaka University Medical School, Japan.

## Abstract

**Images:**


Br. J. Cancer (1994), 69, 1015-1020

© Macmillan Press Ltd., 1994

### Modulation of the mevalonate pathway and cell growth by pravastatin and *d*-limonene in a human hepatoma cell line (Hep G2)

S. Kawata, T. Nagase, E. Yamasaki, H. Ishiguro & Y. Matsuzawa

*Second Department of Internal Medicine, Osaka University Medical School, Osaka 565, Japan.*

**Summary** Modulation of cell growth by a combination of pravastatin [a 3-hydroxy-3-methylglutaryl-coenzyme A (HMG-CoA) reductase inhibitor] and *d*-limonene (an inhibitor of protein isoprenylation) was studied using Hep G2, a human hepatoma-derived cell line. Pravastatin, at 0.1 mM, produced 85% inhibition of cholesterol biosynthesis in Hep G2 cells. The combination of 0.1 mM pravastatin and 1.0 mM *d*-limonene had no further effect on the reduction seen with pravastatin alone. Addition of 0.1 mM pravastatin or 1.0 mM *d*-limonene did not significantly suppress DNA synthesis by the cells, whereas the combination suppressed it to 50% of the control level. Production of m-p21$^{ras}$ was markedly decreased to 35% of the control level by the combination of these two inhibitors. Both the reduction by pravastatin of farnesylpyrophosphate as substrate for protein:farnesyl transferase and inhibition of protein farnesylation by *d*-limonene seem to be responsible for the profound suppression of m-p21$^{ras}$ formation in the cells. However, dolichol synthesis was not suppressed by the combination of these inhibitors. In human fibroblasts, the combination suppressed m-p21$^{ras}$ production but not DNA synthesis. These findings suggest that the combination of pravastatin and *d*-limonene acts on cancer cell growth through inhibition of the post-translational processing of cellular proteins including p21$^{ras}$, rather than through the suppression of cholesterol and dolichol biosynthesis. Thus, the combination of an HMG-CoA reductase inhibitor and an inhibitor of protein isoprenylation offers potential as a new approach for cancer therapy.

The activity of 3-hydroxy-3-methylglutaryl-coenzyme A (HMG-CoA) reductase [mevalonate NADP:oxidoreductase (CoA-acylating), EC 1.1.1.34], the major rate-limiting enzyme in cholesterol biosynthesis, has been suggested to show a positive correlation with DNA synthesis and growth in mammalian cells (Kandutsch & Chen, 1977; Chen, 1981). In addition to serving as a precursor of the structural cholesterol required for cell proliferation, the mevalonic acid produced by HMG-CoA reductase seems to regulate cell growth independent of cholesterogenesis by playing a direct role in DNA synthesis (Quesney-Huneeus *et al*., 1979). Cellular proteins which participate in cell growth regulation, such as Ras p21 and lamins A and B, have recently been shown to undergo covalent modification at the carboxyl terminus by mevalonate-derived farnesyl isoprenoid (Beck *et al*., 1988; Wolda & Glomset, 1988; Casey *et al*., 1989; Hancock *et al*, 1989; Schafer *et al*., 1989; Goldstein & Brown, 1990).

Manipulation of the mechanisms regulating the mevalonate pathway may offer a new form of therapy for certain human cancers. In addition to their cholesterol-lowering activity, HMG-CoA reductase inhibitors (including lovastatin) exhibit a cytostatic effect when added to cultures of proliferating cells (Goldstein *et al*., 1979; Habenicht *et al*., 1980; Fairbanks *et al*., 1984; Maltese, 1984) as well as *in vivo* (Maltese *et al*., 1985). There seem to be three mechanisms that contribute to the effect of HMG-CoA reductase inhibitors on cell growth: (1) a decrease in the cellular cholesterol content owing to inhibition of cholesterol biosynthesis, (2) a reduction of the levels of dolichols and ubiquinones and (3) inhibition of the isoprenylation processing of cellular proteins including Ras proteins.

Limonene, the predominant monoterpene in orange peel oil, has substantial chemopreventive and therapeutic effects against chemically induced cancers in rodents (Elegbede *et al*., 1984; Wattenberg & Coccia, 1991; Crowell, 1992). Recently, *d*-limonene was reported to be a selective inhibitor of the isoprenylation of 21-26 kDa non-nuclear proteins in intact cells (Crowell *et al*., 1991). The farnesyl:protein transferase (FT) that acts on p21$^{ras}$ is a likely target of *d*-limonene, although it has not yet been directly demonstrated that *d*-limonene is an inhibitor of FT. However, cell growth was not suppressed when high concentrations of *d*-limonene were added to fibroblast cultures (Crowell *et al*., 1991). One possible explanation for this finding is that *d*-limonene does not completely block isoprenylation and that subnormal levels of isoprenylation may be sufficient for a cell growing normally (Crowell *et al*., 1991). Thus, the *in vivo* anti-tumour activity of limonene could be caused by the greater dependence of cancer cells on isoprenylated growth control proteins when compared with normal cells (Crowell *et al*., 1991). It might be expected that both reducing farnesyl pyrophosphate (the substrate for FT) by using HMG-CoA reductase inhibitor and inhibiting FT itself with *d*-limonene could strongly suppress the farnesylation of proteins, including p21$^{ras}$, in cancer cells. Thus, it seems worthwhile to examine whether the combination of HMG-CoA reductase inhibitor with *d*-limonene has an augmented effect on protein isoprenylation and the growth of cancer cells.

The Hep G2 cell line derived from a human hepatocellular carcinoma shows overexpression of Ras protein with a point mutation in codon 61 of the N-*ras* gene (Richards *et al*., 1990). The cholesterol metabolism of this cell line has already been studied in detail (Knowles *et al*., 1980; Wu *et al*., 1984; Hoeg *et al*., 1985; Erickson & Fielding, 1986). Thus, the Hep G2 cell line appears to be a good candidate for testing the growth-inhibitory effect of an HMG-CoA reductase inhibitor combined with *d*-limonene. In the present study, to clarify the effect of both inhibitors on cancer cell growth, we examined the changes in cholesterol and dolichol biosynthesis and the changes in the DNA synthesis and membrane-bound isoprenylated Ras p21 (m-p21$^{ras}$) production caused by pravastatin (Tsujita *et al*., 1986; Mosley *et al*., 1989; Reihner *et al*., 1990), a potent HMG-CoA reductase inhibitor, and *d*-limonene in this cell line. In addition, a comparison was made with the changes in DNA synthesis and m-p21$^{ras}$ production in human fibroblasts by a combination of both inhibitors.

### Materials and methods

### *Cell growth assay*

Hep G2 cells and human skin fibroblasts were maintained in Dulbecco's modified Eagle medium (DMEM) with 10% fetal

Correspondence: S. Kawata, Second Department of Internal Medicine, Osaka University Medical School, 2-2 Yamada-oka, Suita-shi, Osaka 565, Japan.

Received 15 July 1993; and in revised form 18 January 1994.

calf serum in an atmosphere of air + 5% carbon dioxide at 37°C. Cultured fibroblasts were derived from skin biopsy from a normal subject. The cells were grown in a monolayer and used between the fifth and 20th passages. To study DNA synthesis, Hep G2 cells and fibroblasts were cultured at a density of $3.2 \times 10^3$ per well in 96-well microplates with DMEM containing 2% bovine serum albumin (BSA), human low-density lipoprotein (LDL, 200 μg of protein per ml) and pravastatin (Sankyo, Tokyo) (0, 0.01, 0.1, 1.0 mM) in the presence or absence of 1 mM *d*-limonene (Aldrich, >99% pure by capillary gas chromatography analysis). After 24 h, [$^3$H]thymidine (1 μCi per well) was added with or without aphidicolin (10 μg ml$^{-1}$). Two hours later, DNA synthesis was assayed by measuring the incorporation of [$^3$H]thymidine. For determination of cell proliferation, Hep G2 cells were cultured at a density of $1.5 \times 10^5$ cells per 60 mm dish with the same medium in the presence of pravastatin and/or *d*-limonene. After 72 h, cell numbers were determined. Each assay was carried out in triplicate.

Human LDL (density 1.019–1.063 g ml$^{-1}$) was obtained from the plasma of healthy subjects and prepared by differential ultracentrifugation according to the method of Brown *et al.* (1974).

*Assay of cholesterol and dolichol biosynthesis*

The rate of cholesterol biosynthesis from [$^{14}$C]acetate was determined by assaying cholesterol digitonide according to a modification of the method of Popjak (1969). Hep G2 cells were cultured for 2 h in 25 cm$^2$ flasks with DMEM containing 2% BSA, human LDL (200 μg of protein per ml), [2-$^{14}$C]acetate (0.5 μCi per flask, New England Nuclear) and pravastatin (0, 0.01, 0.1, or 1.0 mM) in the presence or absence of 1 mM *d*-limonene. The cells were harvested with EDTA (0.02%, w/v) and centrifuged at 1,500 r.p.m. for 10 min. The cell pellet was dissolved in 2 ml of 2 M sodium hydroxide. After saponification and extraction of the non-saponifiable components with petroleum ether, an aliquot of the cholesterol digitonide dissolved in methanol was added to 15 ml of 0.3% 2,5-diphenyloxazole (PPO) and 0.015% bis-phenyloxazolylbenzene solution in toluene, and the $^{14}$C content was determined with a liquid scintillation counter.

The rate of dolichol biosynthesis from [2-$^{14}$C]acetate was assessed by using a modification of the method for separation and determination of dolichol with reversed-phase thin-layer chromatography described by Eggens *et al.* (1983). Hep G2 cells were cultured for 2 h with [2-$^{14}$C]acetate and 0, 0.01, 0.1 or 1.0 mM pravastatin in the presence and absence of 1 mM *d*-limonene. After harvesting of cultures, the cell pellet was dissolved in 2 ml of 2 M sodium hydroxide. Following saponification and extraction of the non-saponifiable components with petroleum ether, the pooled extract was washed with an equal volume of 1 M sodium chloride. Known amounts (2.4 nmol) of dolichol-17 and dolichol-23 (Sigma, St Louis, MO, USA) were added as standards. The petroleum solvent was evaporated with nitrogen and supplemented with 0.5 ml of Lipidex-5000 and 300 μl of water. A Lipidex-5000 column was prepared by placing acetone-treated Lipidex into a Pasteur pipette (about 1 cm). Next, the sample was suspended in Lipidex–acetone–water and was applied to the column. After washing with methanol, the dolichols were eluted with 2.5 ml of chloroform–methanol (2:1, v/v) and were oxidised to alcohols with an oxidising mixture containing chromium oxide, anhydrous pyridine and dichloromethane. After vortexing for 15 s, the reaction was stopped with methanol, and the test dye–lipophilic mixture for lipid chromatography (Merck) was added. The sample was then applied to a silica gel 60 (230–400 mesh, Merck) column (4 × 0.5 cm) and eluted with 1.5 ml of toluene. The polyprenol was collected together with the red dye, and the eluate was evaporated under nitrogen and dissolved in 30 μl of acetone. This sample was applied to a PR-18 HPTLC plate (Merck), which was developed in pure acetone. After staining in iodine, the dolichol bands were scraped off to determine their $^{14}$C content with a liquid scintillation counter.

*Sodium dodecyl sulphate (SDS) gel analysis of post-translational processing of p21$^{ras}$*

Hep G2 cells or fibroblasts were preincubated for 2 h in methionine-free DMEM containing 2% BSA, human LDL (200 μg of protein per ml) and pravastatin (0, 0.01, 0.1, or 1.0 mM) in the presence or absence of 1 mM *d*-limonene or 20 mM mevalonate before incubation with [$^{35}$S]methionine. The cells were then incubated for 12 h with [$^{35}$S]methionine (100 μCi ml$^{-1}$, New England Nuclear) in the same medium. All cells were lysed on ice for 10 min in immunoprecipitation buffer (50 mM Tris–HCl, pH 7.5, 20 mM magnesium chloride, 150 mM sodium chloride and 1% aprotinin) containing 1% Triton X-114 and were centrifuged at 10,000 *g* at 4°C to remove insoluble debris according to a modification of the method of Gutierrez *et al.* (1989). The samples were immunoprecipitated for 16 h at 4°C with an anti-Ras p21 monoclonal antibody (Y13–259, ATCC, 1:50 dilution), using fresh protein A–Sepharose CL-4B beads (Sigma) precoated with rabbit anti-mouse IgG. The precipitated beads were washed twice with washing buffer (50 mM Tris–HCl, pH 7.5, 20 mM magnesium chloride and 150 mM sodium chloride) and suspended in 20 μl of 20 mM Tris–HCl (pH 7.5)–20 mM EDTA – 2% SDS. This suspension was incubated at 100°C for 5 min to elute bound p21$^{ras}$ and was then centrifuged. The immunoprecipitated Ras proteins were analysed by 8–15% gradient SDS-PAGE and the $^{35}$S-labelled proteins on the gels were identified by fluorography. The bands were excised and quantiated by liquid scintillation spectrometry.

[$^{35}$S]Methionine incorporation into p21$^{ras}$ in Hep G2 cells or fibroblasts was at isotopic equilibrium after 11 h of labelling, and there was no change in the specific radioactivity of p21$^{ras}$ between 11 and 14 h under incubation conditions. Therefore, a 12 h labelling period was used to examine the effect of pravastatin on the cellular level of mature p21$^{ras}$.

*Other assays*

The free and esterified cholesterol contents in Hep G2 cells were determined using methods described previously (Kawata *et al.*, 1987, 1990). The protein content of the cells was determined by the method of Lowry *et al.* (1951).

*Statistical analysis*

Statistical analysis was done using Student's *t*-test.

### Results

Pravastatin decreased the rate of cholesterol biosynthesis in Hep G2 cells in a dose-dependent manner in the absence of *d*-limonene in the culture medium (Figure 1). Addition of 1.0 mM *d*-limonene did not alter the degree of suppression of cholesterol biosynthesis by 0.01, 0.1 and 1.0 mM pravastatin. Addition of 1.0 mM pravastatin suppressed cholesterol biosynthesis to less than 10% of the control value both in the presence and absence of *d*-limonene. The cholesterol content was decreased in a dose-dependent manner by the addition of pravastatin to Hep G2 cultures, and the cholesterol content was not altered any further by the addition of *d*-limonene (Figure 2).

DNA synthesis by Hep G2 cells was decreased in a dose-dependent manner by the addition of pravastatin in the absence of *d*-limonene (Figure 3). Pravastatin did not significantly suppress DNA synthesis at 0.1 mM, but suppressed it to approximately 70% of the control value at 1.0 mM. Pravastatin also suppressed the cell number to approximately 30% of the control value at 1.0 mM. The concentration dependence of the inhibition of dolichol synthesis was similar to that for m-p21$^{ras}$, while cholesterol biosynthesis was inhibited by a much lower concentration of

**Figure 1** Effect of pravastatin on cholesterol biosynthesis from [[14]C]acetate in Hep G2 cells in the presence (○) and absence (●) of 1.0 mM *d*-limonene. Each value represents the average of triplicate samples.

**Figure 2** Cholesterol content of Hep G2 cells in the presence (○) and absence (●) of 1.0 mM *d*-limonene. **a**, Total cholesterol content; **b**, Esterified cholesterol content. Each value represents the average of triplicate samples.

pravastatin. The concentration dependence of the suppression of DNA synthesis paralleled that of m-p21[ras] or dolichol synthesis.

DNA synthesis by Hep G2 cells was decreased in a dose-dependent manner by the addition of *d*-limonene in the absence of pravastatin (Figure 4). *d*-Limonene did not significantly suppress DNA synthesis at 1.0–2.0 mM *d*-limonene, while 3.0 and 4.0 mM *d*-limonene suppressed DNA synthesis to approximately 50 and 20%, respectively, of the control value. *d*-Limonene at 3.0 and 4.0 mM suppressed the cell number to approximately 25 and 10%, respectively, of the control value. The concentration dependence of the suppression of DNA synthesis paralleled that of m-p21[ras] synthesis.

The effect of a combination of pravastatin and *d*-limonene on DNA synthesis, m-p21[ras] production and dolichol synthesis was then examined in Hep G2 cells (Figures 5 and 6). Addition of either 0.1 mM pravastatin or 1.0 mM *d*-limonene to the culture medium did not significantly suppress DNA synthesis, whereas a combination of 0.1 mM pravastatin and 1.0 mM *d*-limonene suppressed it to approximately 50% of the control level. Addition of either 0.1 mM pravastatin or 1.0 mM *d*-limonene did not significantly suppress the cell proliferation, whereas the combination suppressed the cell number to approximately 35% of the control value ($1.51 \times 10^5$ per dish *vs* $4.32 \times 10^5$ per dish, the average of triplicate samples). Production of m-p21[ras] was markedly decreased to approximately 35% of the control level by the combination of both inhibitors. In contrast, dolichol synthesis was not significantly suppressed by the combination of both inhibitors.

To test the effect of a combination of pravastatin and *d*-limonene in non-transformed cells, we examined the effect on DNA synthesis and m-p21[ras] production in human fibroblasts (Figure 7). Addition of either 0.1 mM pravastatin or 1.0 mM *d*-limonene to the culture medium did not significantly suppress DNA synthesis in the fibroblasts, while their combination suppressed production of m-p21[ras] but not DNA synthesis.

### Discussion

HMG-CoA reductase inhibitors effectively suppressed cholesterol biosynthesis in mammalian cells (Endo *et al.*, 1976; Alberts *et al.*, 1980). Since mevalonic acid, the product of the reaction catalysed by HMG-CoA reductase, is required for the biosynthesis of a number of cellular isoprenoids (Gough & Hemming, 1970; Martin & Thorne, 1974; Faust *et al.*, 1979; Nambudiri *et al.*, 1980), inhibition of this enzyme can result in suppression of the synthesis of various other compounds. A notable class of endogenously synthesised isoprenoids are the isoprenylated substituents post-translationally incorporated into cellular proteins, such as Ras p21 and lamins (Beck *et al.*, 1988; Wolda & Glomset, 1988; Casey

**Figure 3** Concentration dependence of the inhibition of cholesterol (○) and dolichol (△) biosynthesis, m-p21[ras] formation (●) and DNA synthesis (■) in Hep G2 cells by pravastatin. Each value represents the average of triplicate samples.

**Figure 4** Concentration dependence of the inhibition of m-p21[ras] formation (●) and DNA synthesis (■) in Hep G2 cells by *d*-limonene. Each value represents the average of triplicate samples.

et al., 1989; Hancock et al., 1989; Schafer et al., 1989; Goldstein & Brown, 1990).

Pravastatin, a potent HMG-CoA reductase inhibitor, decreased cholesterol biosynthesis in Hep G2 cells in a dose-dependent manner in the present study. Addition of 0.1 mM pravastatin suppressed cholesterol biosynthesis to 15% of the control level. The concentration at which pravastatin suppressed cholesterol biosynthesis in Hep G2 cells was much higher than those reported previously for lovastatin and simvastatin (Shaw et al., 1990).

The dramatic effect of pravastatin on cholesterol biosynthesis in Hep G2 cells appeared to be inconsistent with the fairly modest effects on cellular contents of total and esterified cholesterol. However, several investigations *in vitro* and *in vivo* showed that HMG-CoA reductase inhibitors including pravastatin can induce binding activity of LDL receptors and thus supply exogenous cholesterol to the cholesterol-depleted cells (Brown et al., 1981; Kovanen et al., 1981; Reihner et al., 1990). It is likely that the Hep G2 cells treated with pravastatin could enhance the uptake of LDL-cholesterol in the medium through an increased binding activity of LDL receptors, although the binding activity was not examined in this study.

Inhibition of the formation of m-p21$^{ras}$ and dolichols by pravastatin occurred at similar concentrations in Hep G2 cells, whereas cholesterol biosynthesis was inhibited at a much lower concentration. Sinensky et al. (1990) studied a Chinese hamster ovarian cell line and HeLa cells and demonstrated that the degree of inhibition of HMG-CoA reductase by lovastatin that was required to completely block the formation of m-p21$^{ras}$ and lamin A was much greater than that required for 50% inhibition of cholesterol synthesis. In this study, we found that the pravastatin concentration inhibiting dolichol biosynthesis was similar to that inhibiting the formation of m-p21$^{ras}$. A possible explanation for the relative insensitivity of isoprenylated protein synthesis to the inhibition of mevalonate biosynthesis is that the $K_m$ for the farnesyl-pyrophosphate substrate of prenyl transferase is substantially lower than that for squalene synthase (Sinensky et al., 1990). It has been reported that, in the case of the prenyl transferase, which forms dolichol pyrophosphate, the $K_m$ value for farnesylphosphate is lower than that for squalene synthase, thus allowing the synthesis of isoprenoids despite the partial inhibition of mevalonate biosynthesis (James & Kandutsch, 1979).

DNA synthesis in Hep G2 cells was suppressed in parallel with the inhibition of m-p21$^{ras}$ formation by 0.01, 0.1 and

| | | | | | |
|---|---|---|---|---|---|
| Pravastatin (mM) | 0 | 0.1 | 1.0 | 0.1 | 1.0 |
| d-Limonene (mM) | 0 | 0 | 0 | 1.0 | 0 |
| Mevalonate (mM) | 0 | 0 | 0 | 0 | 20 |

**Figure 5** SDS gel analysis of the effect of a combination of pravastatin and *d*-limonene on the post-translational processing of Ras p21 protein in Hep G2 cells. p, pro-p21$^{ras}$, m, m-p21$^{ras}$.

**Figure 6** Effect of a combination of pravastatin and *d*-limonene on DNA synthesis, m-p21$^{ras}$ formation and dolichol biosynthesis in Hep G2 cells. P, 0.1 mM pravastatin; L, 1.0 mM *d*-limonene; P + L, 0.1 mM pravastatin plus 1.0 mM *d*-limonene. Mean values of triplicate cultures ± s.e. are shown for each condition.

**Figure 7** Effect of a combination of paravastatin and *d*-limonene on DNA synthesis and m-p21$^{ras}$ formation in human fibroblasts. P, 0.1 mM pravastatin; L, 1.0 mM *d*-limonene, P + L, 0.1 mM pravastatin plus 1.0 mM *d*-limonene. Mean values of triplicate cultures ± s.e. are shown for each condition.

1.0 mM pravastatin. This observation raises the possibility that the growth inhibition of Hep G2 cells by pravastatin might have been related to suppression of isoprenylation of the activated N-Ras protein with a point mutation, although there is still no direct evidence that N-*ras* activation is required for the growth of these cells. Mendola and Backer (1990) showed that N-*ras* oncogene-induced neuronal differentiation of UR61J rat phaeochromocytoma cells is blocked by lovastatin. However, Declue *et al.* (1991) demonstrated, using a series of NIH3T3 cell lines transformed by oncogenes, including v-*ras* and c-*ras*, that inhibition of cell growth by lovastatin was not specific for cells in which transformation was dependent upon isoprenylated Ras proteins. Thus, the HMG-CoA reductase inhibitors seem to modulate diverse cellular functions through their suppression of isoprenoid formation and thus inhibit cell growth.

In this study, we examined the inhibitory effect of the combination of pravastatin and *d*-limonene, a selective inhibitor of protein isoprenylation, on the mevalonate pathway and cell growth. The combination of 0.1 mM pravastatin and 1.0 mM *d*-limonene suppressed DNA synthesis to approximately 50% of the control level in Hep G2 cells, whereas 0.1 mM pravastatin and 1.0 mM *d*-limonene alone did not significantly suppress DNA synthesis. The combination of pravastatin and *d*-limonene had no further effect on the reduction of cholesterol biosynthesis seen with pravastatin alone. This finding agreed with the report of Crowell *et al.* (1991) that a combination of *d*-limonene and simvastatin, an HMG-CoA reductase inhibitor, had no further effect on reduction of cholesterol biosynthesis in NIH3T3 cells seen with simvastatin alone. In addition, the combination of 0.1 mM pravastatin and 1 mM *d*-limonene did not significantly suppress dolichol biosynthesis by Hep G2 cells. These observations suggest that the inhibitory effect of the combination of 0.1 mM pravastatin and 1.0 mM *d*-limonene on the growth of Hep G2 cells was not derived from the depletion of cellular cholesterol or the inhibition of dolichol biosynthesis.

The combination of pravastatin and *d*-limonene suppressed m-p21$^{ras}$ formation to less than 50% of the control level, whereas pravastatin or *d*-limonene alone did not significantly affect it. HMG-CoA reductase inhibitors suppress the production of farnesylpyrophosphate and geranylgeranylpyrophosphate, which are isoprenylated substitutents that are post-translationally incorporated into cellular proteins including Ras p21 and lamins. On the other hand, Crowell *et al.* (1991) showed that *d*-limonene does not affect the prenylation of nuclear proteins including lamins, but instead selectively affects the prenylation of 21–26 kDa non-nuclear proteins including Ras p21. Thus, both the reduction of farnesylpyrophosphate as substrate for FT and inhibition of FT itself by *d*-limonene seem to be responsible for the profound suppression of the formation of m-p21$^{ras}$ in Hep G2 cells. Very recent studies demonstrated that lamin processing is less sensitive than Ras p21 processing to inhibition by known inhibitors of FT (Garcia *et al.*, 1993; James *et al.*, 1993).

The combination of both inhibitors suppressed m-p21$^{ras}$ production in human fibroblasts but not DNA synthesis. Pravastatin decreased the rate of cholesterol biosynthesis in human fibroblasts in a similar manner to that in Hep G2 cells, and the combination of both inhibitors had no further effect on the reduction in the fibroblasts (data not shown). Our observations suggest that the reduction of protein isoprenylation by the combination of both inhibitors suppressed DNA synthesis in cancer cells but not in non-transformed cells. This implies that subnormal levels of isoprenylated growth control proteins may be sufficient for the proliferation of non-transformed cells. The combination of an HMG-CoA reductase inhibitor and an inhibitor of protein isoprenylation may offer a new approach to cancer chemotherapy.

### References

ALBERTS, A.W., CHEN, J., KURON, J., HUNT, V., HUFF, J., HOFFMAN, C., ROTHROCK, J., LOPEZ, M., JOSHUA, H., HARRIS, E., PATCHETT, A., MONAGHAN, R., CURRIE, S., STAPLEY, E., ALBERS-SCHONBERG, G., HENSENS, O., HIRSCHFIELD, J., HOOGSTEEN, K., LIESCH, J. & SPRINGER, J. (1980). Mevinolin: a highly potent competitive inhibitor of hydroxymethylglutaryl-coenzyme A reductase and a cholesterol-lowering agent. *Proc. Natl Acad. Sci. USA*, **77**, 3957–3961.

BECK, L., HOSICK, T.J. & SHINENSKY, M. (1988). Incorporation of a product of mevalonic acid metabolism into proteins of Chinese hamster ovary cell nuclei. *J. Cell Biol.*, **107**, 1307–1316.

BROWN, M.S. & GOLDSTEIN, J.L. (1974). Familiar hypercholesterolemia: defective binding of lipoproteins to cultured fibroblasts associated with impaired regulation of 3-hydroxy-3-methylglutaryl coenzyme A reductase activity. *Proc. Natl Acad. Sci. USA*, **71**, 788–792.

BROWN, M.S., KOVANEN, P.T. & GOLDSTEIN, J.L. (1981). Regulation of plasma cholesterol by lipoprotein receptors. *Science*, **212**, 628–635.

CASEY, P.J., SOLSKI, P.A., DER, C.J. & BUSS, J.E. (1989). p21 *ras* is modified by a farnesyl isoprenoid. *Proc. Natl Acad. Sci. USA*, **86**, 8323–8327.

CHEN, H.W. (1981). The activity of 3-hydroxy-3-methylglutaryl coenzyme A reductase and the rate of sterol synthesis diminish in cultures with high cell density. *J. Cell Physiol.*, **108**, 91–97.

CROWELL, P.L., CHANG, R.R., REN, Z., ELSON, C.E. & GOULD, M.N. (1991). Selective inhibition of isoprenylation of 21–26 kDa proteins by the anticarcinogen *d*-limonene and its metabolites. *J. Biol. Chem.*, **266**, 17697–17685.

CROWELL, P.L., KENNAN, W.S., HAAG, J.D., AHMAD, S., VEDEJS, E. & GOULD, M.N. (1992). Chemoprevention of mammary carcinogenesis by hydroxylated derivatives of *d*-limonene. *Carcinogenesis*, **13**, 1261–1264.

DECLUE, J.E., VASS, W.C., PAPAGEORGE, A.G., LOWRY, D.R. & WILLUMSEN, B.M. (1991). Inhibition of cell growth by lovastatin is independent of *ras* function. *Cancer Res.*, **51**, 712–717.

EGGENS, I., CHOJNACKI, T., KENNE, L. & DALLNER, G. (1983). Separation, quantitation and distribution of dolichol and dolichyl phosphate in rat and human tissues. *Biochim. Biophys. Acta*, **751**, 355–368.

ELEGBEDE, J.A., ELSON, C.E., QURESHI, A., TANNER, M.A. & GOULD, M.N. (1984). Inhibition of DMBA-induced mammary cancer by the monoterpene *d*-limonene. *Carcinogenesis*, **5**, 661–664.

ENDO, A., KURODA, M. & TANZAWA, K. (1976). Competitive inhibition of 3-hydroxy-3-methylglutaryl coenzyme A reductase by ML-236A and ML-236B, fungal metabolites having hypocholesterolemic activity. *FEBS Lett.*, **72**, 323–326.

ERICKSON, S.K. & FIELDING, P.E. (1986). Parameters of cholesterol in the human hepatoma cell line, Hep G2. *J. Lipid Res.*, **27**, 875–883.

FAIRBANKS, K.P., WITTE, L.D. & GOODMAN, D.S. (1984). Relationship between mevalonate and mitogenesis in human fibroblasts stimulated with platelet-derived growth factor. *J. Biol. Chem.*, **259**, 1546–1551.

FAUST, J.R., GOLDSTEIN, J.L. & BROWN, M.S. (1979). Synthesis of ubiquinone and cholesterol in human fibroblasts: regulation of a branched pathway. *Arch. Biochem. Biophys.*, **192**, 86–99.

FAUST, J.R., BROWN, M.S. & GOLDSTEIN, J.L. (1980). Synthesis of 2-isopentenyl tRNA from mevalonate in cultured human fibroblasts. *J. Biol. Chem.*, **255**, 6546–6548.

GARCIA, A.M., ROWELL, C., ACKERMANN, K., KOWALCZYK, J.J. & LEWIS, M.D. (1993). Peptidomimetic inhibitors of Ras farnesylation and function in whole cells. *J. Biol. Chem.*, **268**, 18415–18418.

GOLDSTEIN, J.L. & BROWN, M.S. (1990). Regulation of the mevalonate pathway. *Nature*, **343**, 425–430.

GOLDSTEIN, J.L., HELGESON, J.A.S. & BROWN, M.S. (1979). Inhibition of cholesterol synthesis with compactin renders growth of cultured cells dependent on the low density lipoprotein receptors. *J. Biol. Chem.*, **254**, 5403–5409.

GOUGH, D.P. & HEMMING, F.W. (1970). The characterization and stereochemistry of biosynthesis of dolichols in rat liver. *Biochem. J.*, **118**, 163–166.

GUTIERREZ, L., MAGEE, A.I., MARSHALL, C.J. & HANCOCK, J.F. (1989). Post-translational processing of p21$^{ras}$ is two-step and involves carboxy-terminal proteolysis. *EMBO J.*, **8**, 1093–1098.

HABENICHT, A.J.R., GLOMSET, J.A. & ROSS, R. (1980). Relation of cholesterol and mevalonic acid to the cell cycle in smooth muscle and Swiss 3T3 cells stimulated to divide by platelet-derived growth factor. *J. Biol. Chem.*, **255**, 5134–5140.

HANCOCK, J.F., MAGEE, A.I., CHILDS, J.E. & MARSHALL, C.J. (1989). All *ras* proteins are polyisoprenylated but only some are palmitoylated. *Cell*, **57**, 1167–1177.

HOEG, J.M., DEMOSKY, Jr, S.J., EDGE, S.B., GREGG, R.E., OSBORNE, J.C. & BREWER, Jr, H.B. (1985). Characterization of a human hepatic receptor for high density lipoproteins. *Arterioschlerosis*, **5**, 228–237.

JAMES, G.L., GOLDSTEIN, J.L., BROWN, M.S., RAWSON, T.E., SOMERS, T.C., MCDOWELL, R.S., CROWLEY, C.W., LUCAS, B.K., LEVINSON, A.D. & MARSTERS, Jr, J.C. (1993). Benzodiazepine peptidomimetics: potent inhibitors of Ras farnesylation in animal cells. *Science*, **260**, 1937–1942.

JAMES, M.J. & KANDUTSCH, A.A. (1979). Inter-relationship between dolichol and sterol synthesis in mammalian cell cultures. *J. Biol. Chem.*, **254**, 8442–8446.

KANDUTSCH, A.A. & CHEN, H.W. (1977). Consequences of blocked sterol synthesis in cultured cells, DNA synthesis and membrane composition. *J. Biol. Chem.*, **252**, 409–415.

KAWATA, S., CHITRANUKROH, A., OWEN, J.S. & MCINTYRE, N. (1987). Membrane lipid changes in erythrocytes, liver and kidney in acute and chronic experimental liver disease in rats. *Biochim. Biophys. Acta*, **896**, 26–34.

KAWATA, S., TAKAISHI, K., NAGASE, T., ITO, N., MATSUDA, Y., TAMURA, S., MATSUZAWA, Y. & TARUI, S. (1990). Increase in the active form of 3-hydroxy-3-methylglutaryl coenzyme A reductase in human hepatocellular carcinoma: possible mechanism for alteration of cholesterol biosynthesis. *Cancer Res.*, **50**, 3270–3273.

KNOWLES, B.B., HOWE, C.C. & ADEN, D.P. (1980). Human hepatocellular carcinoma cell lines secrete the major plasma proteins and hepatitis B surface antigen. *Science*, **209**, 497–499.

KOVANEN, P.T., BILHEIMER, D.W., GOLDSTEIN, J.L., JARAMILLO, J.J. & BROWN, M.S. (1981). Regulation role for hepatic low density lipoprotein receptors *in vivo* in the dog. *Proc. Natl Acad. Sci. USA*, **78**, 1194–1198.

LOWRY, O.H., ROSEBROUGH, N.J., FARR, A.J. & RANDALL, R.J. (1951). Protein measurement with the Folin phenol reagent. *J. Biol. Chem.*, **193**, 265–275.

MALTESE, W.A. (1984). Induction of differentiation in murine neuroblastoma cells by mevinolin, a competitive inhibitor of 3-hydroxy-3-methylglutaryl coenzyme A reductase. *Biochem. Biophys. Res. Commun.*, **120**, 454–460.

MALTESE, W.A., DEFENDINI, R., GREEN, R.A., SHERIDAN, K.M. & DONLEY, D.K. (1985). Suppression of murine neuroblastoma growth *in vivo* by mevinolin, a competitive inhibitor of 3-hydroxy-3-methylglutaryl coenzyme A reductase. *J. Clin. Invest.*, **76**, 1748–1754.

MARTIN, H.G. & THORNE, K.J.I. (1974). Synthesis of radioactive dolichol from (4S-$^{3}$H) mevalonate in the regenerating rat liver. *Biochem. J.*, **138**, 277–280.

MENDOLA, C.E. & BACKER, J.M. (1990). Lovastatin blocks N-*ras* oncogene-induced neuronal differentiation. *Cell Growth Different.*, **1**, 499–502.

MOSLEY, S.T., KALINOWSKI, S.S., SCHAFER, B.L. & TANAKA, R.D. (1989). Tissue-selective acute effects of inhibitors of 3-hydroxy-3-methylglutaryl coenzyme A reductase on cholesterol biosynthesis in lens. *J. Lipid Res.*, **30**, 1411–1419.

NAMBUDIRI, A.M., RANGANATHAN, D.S. & RUDNEY, H. (1980). The role of 3-hydroxy-3-methylglutaryl coenzyme A reductase activity in the regulation of ubiquinone synthesis in human fibroblasts. *J. Biol. Chem.*, **255**, 5894–5899.

POPJAK, G. (1969). Enzymes of sterol biosynthesis. *Methods Enzymol.*, **15**, 393–454.

QUESNEY-HUNEEUS, V., WHILY, M.H. & SIPERSTEIN, M.D. (1979). Essential role for mevalonate synthesis in DNA replication. *Proc. Natl Acad. Sci. USA*, **76**, 5056–5060.

REIHNER, E., RUDLING, M., STAHLBERG, D., BERGLUND, L., EWERTH, S., BJKORKHEM, I., EINARSSON, K. & ANGELIN, B. (1990). Influence of pravastatin, a specific inhibitor of·HMG-CoA reductase, on hepatic metabolism of cholesterol. *N. Engl. J. Med.*, **323**, 224–228.

RICHARDS, C.A., SHORT, S.A., THORGEIRSSO, S.S. & HUBER, B.E. (1990). Characterization of a transforming N-*ras* gene in the human hepatoma cell line Hep G2: additional evidence for the importance of c-*myc* and *ras* cooperation in hepatocarcinogenesis. *Cancer Res.*, **50**, 1521–1527.

SCHAFER, W.R., KIM, R., STERNE, R., THORNER, J., KIM, S.-H. & RINE, J. (1989). Genetic and pharmacological suppression of oncogenic mutations in *ras* genes of yeast and humans. *Science*, **245**, 379–385.

SHAW, M.K., NEWTON, R.S., SLISKOVIC, D.R., ROTH, B.D., FERGUSON, E. & KRAUSE, B.R. (1990). Hep-G2 cells and primary hepatocytes differ in their response to inhibitors of HMG-CoA reductase. *Biochem. Biophys. Res. Commun.*, **170**, 726–734.

SINENSKY, M., BECK, L.A., LEONARD, S. & EVANS, R. (1990). Differential inhibitory effects of lovastation on protein isoprenylation and sterol synthesis. *J. Biol. Chem.*, **265**, 19937–19941.

TSUJITA, Y., KURODA, M., SHIMADA, Y., TANZAWA, K., ARAI, M., KANEKO, I., TANAKA, M., MATSUDA, H., TARUMI, C., WATANABE, Y. & FUJII, S. (1986). CS-514, a competitive inhibitor of 3-hydroxy-3-methylglutaryl coenzyme A reductase: tissue-selective inhibition of sterol synthesis and hypolipidemic effect on various animal species. *Biochim. Biophys. Acta*, **877**, 50–60.

WATTENBERG, L.W. & COCCIA, J.B. (1991). Inhibition of 4-(methylnitrosamino)-1-(3-pyridyl)-1-butane. *Carcinogenesis*, **12**, 115–117.

WOLDA, S.L. & GLOMSET, J.A. (1988). Evidence for modification of lamin B by a product of mevalonic acid. *J. Biol. Chem.*, **263**, 5997–6000.

WU, G.Y., WU, C.H., RIFICI, V.A. & STOCKERT, R.J. (1984). Activity and regulation of low density lipoprotein receptors in a human hepatoblastoma cell line. *Hepatology*, **4**, 1190–1194.